# IDH3-dependent mitochondrial function in stromal fibroblasts suppresses malignant tumor growth

**DOI:** 10.1016/j.neo.2026.101361

**Published:** 2026-09-04

**Authors:** Lina Rochelle Koseki, Minoru Kobayashi, Mai Ito, Yoko Nishiga, Tamaki Saito, Shunji Haruna, Atsushi Shibata, Hiroshi Harada

**Affiliations:** a Laboratory of Cancer Cell Biology, Graduate School of Biostudies, Kyoto University, Kyoto, 606-8501, Japan; b Department of Genome Repair Dynamics, Radiation Biology Center, Graduate School of Biostudies, Kyoto University, Kyoto, 606-8501, Japan; c Department of Radiation Oncology and Image-applied Therapy, Graduate School of Medicine, Kyoto University, Kyoto, 606-8507, Japan; d Division of Molecular Oncological Pharmacy, Faculty of Pharmacy, Keio University, Tokyo, 105-8512, Japan

**Keywords:** Cancer, Tumor-infiltrating fibroblast, Mitochondria, TCA cycle, Isocitrate dehydrogenase 3 (IDH3)

## Abstract

Malignant solid tumors comprise not only cancer cells but also diverse non-cancerous stromal cells that shape the tumor microenvironment. The tricarboxylic acid (TCA) cycle has an overarching presence in providing substrates needed to drive the electron transport chain and, ultimately, ATP synthesis. However, it remains unclear which stromal cell lineages influence tumor growth through TCA-dependent mitochondrial function, and whether such activities act in a tumor-promoting or tumor-suppressive manner. Isocitrate dehydrogenase 3 (IDH3), a rate-limiting TCA cycle enzyme that generates NADH to support mitochondrial respiration, provides a genetic entry point to interrogate mitochondrial TCA-dependent function in stromal cells. In this study, we established a mouse model in which tamoxifen administration induces CreER^T2^-dependent knockout of the α subunit of IDH3 (IDH3α) in all somatic cells. Using this model with transplantation of *Idh3a*-intact murine cancer cells, we found that host *Idh3a* deficiency accelerated growth of murine MC38 tumors in a cancer cell line-dependent manner. Bone marrow chimera experiments indicated that hematopoietic lineages were not responsible for this phenotype, suggesting a contribution from tissue-resident non-hematopoietic stromal cells that are not replaced by bone marrow transplantation. Single-cell RNA sequencing of human tumor specimens revealed broad *IDH3A* expression across multiple tumor microenvironment compartments, including fibroblasts. Consistently, *in vitro* co-culture assays demonstrated that *Idh3a*-intact, but not *Idh3a*-KO, fibroblasts suppressed cancer cell proliferation in a contact-dependent manner. Together, these findings identify IDH3α-dependent mitochondrial function in fibroblasts as a critical determinant of tumor progression and suggest that stromal mitochondrial metabolism represents an important axis for modulating tumor behavior.

## Introduction

Malignant solid tumors are not composed solely of cancer cells. A wide variety of non-malignant cells, including fibroblasts, vascular cells, inflammatory cells, and immune cells, coexist within tumors, forming an extremely heterogeneous and complex cellular society [[1], [2], [3]]. This tumor microenvironment has emerged not merely as a factor affecting the structural milieu but as a key determinant of tumor initiation, progression, and therapeutic response [4,5]. Indeed, the proliferative capacity and malignant phenotypes of cancer cells are dictated by both their intrinsic genetic alterations and interactions with neighboring stromal cells. These insights underscore the concept that the tumor microenvironment should be regarded not as a secondary and passive bystander but as a central focus of cancer biology and a potential therapeutic target. However, the identities and characteristics of non-malignant cells that influence cancer cell proliferation have not yet been fully elucidated.

Among intracellular organelles, mitochondria are widely regarded as master regulators of cellular function, coupling metabolic activity to cell fate determination [6,7]. In addition to producing the bulk of the cellular energy currency, ATP, mitochondria coordinate redox balance, biosynthetic metabolism, and stress responses for sustaining tissue homeostasis. In cancer biology, extensive efforts have focused on how mitochondrial metabolism in cancer cells supports rapid proliferation and survival under adverse conditions. However, far less attention has been paid to the functional significance of mitochondrial activity in non-malignant stromal cells within the tumor microenvironment, despite their intimate and dynamic interactions with cancer cells.

Cellular energy production from glucose is orchestrated through three sequential metabolic processes: glycolysis, the tricarboxylic acid (TCA) cycle, and the electron transport chain (ETC), with the TCA cycle serving as a central hub that links carbon metabolism to mitochondrial function. Although glycolysis can generate ATP, its energetic yield is limited, and most cellular processes rely on mitochondrial oxidative phosphorylation driven by NADH mainly supplied from the TCA cycle. Thus, TCA cycle activity represents a critical determinant of mitochondrial functional capacity beyond its role in supporting bulk ATP production. Within the TCA cycle, isocitrate dehydrogenase 3 (IDH3) occupies a unique position as a rate-limiting enzyme that generates NADH, linking TCA cycle flux to ETC activity [8,9]. Unlike IDH1 and IDH2, which produce NADPH and function outside the mitochondrial matrix or in redox homeostasis, IDH3 directly supports mitochondrial respiration [10,11]. IDH3 forms a heterotetramer composed of two α subunits, one β subunit, and one γ subunit, and its enzymatic activity critically depends on IDH3α [12,13]. Accordingly, genetic ablation of *Idh3a* provides a strategy to impair mitochondrial TCA-dependent function *in vivo*, without directly targeting cancer cells themselves [8,9].

Based on this rationale, we reasoned that transplantation of *Idh3a*-intact cancer cells into *Idh3a*-deficient host mice would preserve mitochondrial metabolism in tumor cells, while selectively perturbing mitochondrial function in non-malignant stromal cells within the tumor microenvironment. This genetic strategy allowed us to address a fundamental and previously unresolved question in cancer biology: which non-malignant stromal cell populations rely on mitochondrial function to regulate tumor growth, and whether such mitochondrial dependencies act in a tumor-promoting or tumor-suppressive manner.

In this study, we generated tamoxifen-inducible *Idh3a* conditional knockout (cKO) mice and examined how loss of *Idh3a* in non-malignant stromal cells influences tumor growth. Using a combination of *in vivo* tumor transplantation, bone marrow chimera experiments, single-cell transcriptomic analyses, and *in vitro* co-culture assays, we identify fibroblasts as a key stromal population in which IDH3α-dependent mitochondrial function suppresses tumor cell proliferation.

## Materials and methods

### Genetically modified mice

The targeting vector for generating *Idh3a* knockout (KO) mice was obtained from the European Conditional Mouse Mutagenesis Program (EUCOMM) and microinjected into C57BL/6 J mouse embryonic stem cells (performed by Transgenic, Inc., Hyogo, Japan). Chimeric mice were then generated, and candidate clones harboring the targeted allele were screened to establish heterozygous *Idh3a* KO mice. Targeted integration at the *Idh3a* locus on chromosome 9 was verified by PCR using the following primer sets: NeoRm primer: 5′-GATGGATTGCACGCAGGTTC-3′ and R6-7 intron primer: 5′-TCTTAGGCTAGGGAAGCCGT-3′ to detect the transgene insertion. To address heterozygosity, PCR genotyping was additionally performed using Idh3a_F primer: 5′-TGCTGGGGAGAGGTTTTGTAG-3′ and Idh3a_R primer: 5′-ACCGACAAGAGACACACCTG-3′. Amplification with this primer set yields a 653 bp PCR product from the wild-type allele, whereas the targeted allele generated by the conventional knockout produces a 7,557 bp fragment that cannot be amplified under standard genotyping PCR conditions employing a 1.5 min elongation time. Consequently, this PCR selectively detects the presence of the wild-type *Idh3a* allele. By combining the results of this assay with PCR genotyping using the NeoRm and R6–7 intron primers to detect the targeted allele, heterozygous *Idh3a* knockout mice were reliably identified. The conventional *Idh3a* KO mice were subsequently crossed with CAG-FlpE transgenic (Tg) mice (C57BL/6-Tg(CAG-flpe)36Ito/ItoRbrc, Riken Bio Resource Center), which systemically express a variant of the FLP recombinase (FLPe), to establish *Idh3a* floxed mice. The *Idh3a* floxed mice were further crossed with ROSA26-CreER^T2^ mice (B6.ROSA26Tm(SAEiCE)16Card, Riken Bio Resource Center), which ubiquitously express a tamoxifen-inducible Cre recombinase, ER^T2^iCreER^T2^, to create *Idh3a* cKO mice (*Idh3a*^fl/fl^; CreER^T2+/+^). PCR genotyping of progeny was performed using Idh3a_F and R6-7_intron primers (see above), producing PCR products of 1,160 bp (wild-type), 1,334 bp (floxed), and 653 bp (KO). The ER^T2^iCreER^T2^ insertion at the ROSA26 locus was confirmed by PCR genotyping using iCreF primer: 5′-TTAGCCTTTAAGCCTGCCCA-3′ and iCreR primer: 5′-CCGGGATGCAGAAATTGATGA-3′, yielding a 515 bp product. To address heterozygosity of the ROSA26 locus, PCR genotyping was additionally performed using Rosa26_F primer: 5′-AAAGTCGCTCTGAGTTGTTAT-3′ and Rosa26_R primer: 5′-GGAGCGGGAGAAATGGATATG-3′. Amplification with this primer set yields a 603 bp PCR product from the wild-type allele, whereas the allele with ER^T2^iCreER^T2^ insertion produces a long DNA fragment that cannot be amplified under standard genotyping PCR conditions employing a 1.5 min elongation time. Consequently, this PCR selectively detects the presence of the wild-type ROSA26 allele. By combining these results with PCR genotyping using the iCre_F and iCre_R primers, heterozygous ROSA26-CreER^T2^ mice were reliably identified. The cKO mice (*Idh3a*^fl/fl^; CreER^T2+/+^) were subsequently crossed with C57BL/6-Tg(CAG-EGFP) mice (Japan SLC, Inc.) to generate GFP-expressing cKO mice (*Idh3a*^fl/fl^; CreER^T2+/+^; Tg(CAG-EGFP)) (herein, cKO-GFP) and fluorescence was imaged using IVIS Lumina II (Caliper Life Sciences). Tamoxifen (Sigma-Aldrich, Cat. #T5648) was dissolved in corn oil (Sigma-Aldrich, Cat. #C8267) at 20 mg/mL and administered intraperitoneally at 100 mg/kg daily for 5 consecutive days. Mice were allowed a one-week interval for recovery and to ensure efficient Cre-loxP-mediated knockout prior to subsequent experiments. All mice were maintained under specific pathogen-free conditions at the Institute of Laboratory Animals, Graduate School of Medicine, Kyoto University under a 14-h light/10-h dark cycle with ad libitum access to food.

### Cell culture

MC38 murine colorectal carcinoma cells were kindly provided by Dr. Kenji Chamoto, B16 murine melanoma cells were purchased from American Type Culture Collection, and HEK293TN cells were obtained from System Biosciences. *In vitro* experiments utilized Dulbecco’s Modified Eagle Medium (DMEM; 4.5 g/L-Glucose with L-glutamine and sodium pyruvate; Nacalai Tesque Cat. #08458-16) supplemented with 10% heat-inactivated fetal bovine serum (FBS; Biosera, Cat. #FB-1003/500) and 1% Penicillin-Streptomycin Solution (× 100) (Fujifilm Wako, Cat. #168-23191). To prepare for *in vivo* allografting, MC38 cells were maintained in standard RPMI 1640 media (Nacalai Tesque Cat. #16970-65), supplemented with 10% FBS and 1% penicillin-streptomycin. All cells were cultured at 37°C in a humidified incubator with 95% air and 5% CO_2_. All cell lines were routinely tested for mycoplasma contamination using PCR-based assays and were confirmed to be mycoplasma-free. Cell line authentication was performed by the original suppliers, and cells were used for experiments within a limited number of passages after thawing.

### Plasmid constructs and lentiviruses

To construct the pCDH/EF1α-mIDH3A-myc-His A-IRES-Puro plasmid, cDNA encoding the murine *Idh3a* gene was amplified from mRNA of NIH3T3 cells using mIdh3a_EcoRI_F primer: 5′-GTGGAATTCACCATGGCCGGGTCCGCGTGGG-3′ and mIdh3a_XhoI_R primer: 5′-CGTAGAGTCAAAGACTTAGATCTCGAGTCT-3′ with the LA PCR KIT AMV Ver 1.1 (Takara Bio, Cat. #RR012A). The cDNA was inserted between EcoRI and XhoI of pcDNA4/myc-His A purchased from Thermo Fisher Scientific. Subsequently, the DNA fragment encoding the fusion protein, mouse *Idh3a*-myc-6 × His, was inserted into SwaI and EcoRI sites of pCDH/EF1α-MCS-IRES-Puro vector (System Biosciences, Cat. #CD532A-2). For the immortalization of primary murine tail tip fibroblasts, pCDH/EF1α-HPV18-URR-E6-E7 was used. This plasmid was constructed by inserting cDNA encoding HPV18 E6 and E7, which was prepared by reverse transcription from mRNA of HeLa cells and amplified by PCR using HPV18_EcoRI_F primer: 5′-GCGGAATTCAGATCAATATCCCCTTGGACG-3′ and HPV18_SalI_R primer: 5′-GCAGTCGACTTGCTTACTGCTGGGATGCAC-3′, into EcoRI and SalI sites of pCDH/EF1α-MCS-IRES-Puro vector. To produce lentivirus for the establishment of stable transfectants expressing gene of interest, viral packaging protein coding plasmids, pVSV/G, pPACKH1/GAG, and pPACKH1/REV, were purchased from System Biosciences (Cat. #LV500A-1). To construct pCDH/EF1α-Luc, the DNA fragment encoding firefly luciferase was amplified from the pGL3 Promoter Vector (Promega, Cat. #E1761) by PCR using primers containing NheI and SalI restriction sites (Nfluc_NheI_F primer: 5′-ATAGCTAGCGCCACCATGGAAGACGCC-3′ and Cfluc_SalI_R primer: 5′-ATAGTCGACTTACACGGCGATCTTTCCGCCC-3′), and the resulting PCR product was inserted into the corresponding sites of the pCDH/EF1α-MCS-IRES-Puro vector after removal of the IRES-Puro sequence by NheI and SalI digestion. All lentiviruses were generated using HEK293TN cells through co-transfection with pCDH/EF1α-mIDH3A-Puro-myc-His A or pCDH/EF1α-HPV18-URR-E6-E7 and packaging plasmids (pVSV/G, pPACKH1/GAG, and pPACKH1/REV), as previously described [14]. Empty vector control lentiviruses were prepared using pCDH/EF1α-MCS-IRES-Puro.

### Establishment of *Idh3a* KO fibroblasts

Primary fibroblasts were isolated from tail tip tissues of an 8-week-old *Idh3a*^fl/fl^; CreER^T2+/+^female mouse. The cells were then immortalized by infection with lentivirus expressing HPV E6 and E7, which was prepared by using HEK293TN cells and pCDH/EF1α-HPV18-URR-E6-E7. *Idh3a* knockout was induced by treating the immortalized cells with 5 μM 4-hydroxytamoxifen (4-OHT; Sigma, Cat. #H7904). *Idh3a* KO clones were isolated, and their *Idh3a* knockout was initially confirmed by PCR genotyping using the following primer sets; Idh3a_F primer: 5′-TGCTGGGGAGAGGTTTTGTAG-3′ and R6-7_intron primer: 5′- TCTTAGGCTAGGGAAGCCGT-3′. The genomic DNA purified from the candidate clones was subjected to PCR using Idh3a_F primer and mIDH3a_flox_seq primer: 5′- GTGTAGGCACATAGGGACCA-3′, and the resulting PCR products were purified and subjected to Sanger sequencing using the R6-7_intron primer to verify Cre-mediated deletion of the floxed exon 6 region (Supplementary Fig. 1). Homozygous knockout was further confirmed by the presence of clean single-peak chromatograms in the Sanger sequencing analysis, indicating the absence of residual wild-type alleles. *Idh3a* KO fibroblasts re-expressing IDH3α (*Idh3a*-reconstituted cells) were generated by infection with lentivirus expressing IDH3α and selected with 1 μg/mL puromycin (Nacalai Tesque, Cat. #19752-22).

### Western blot analysis

Whole cell lysates were prepared using Cell Lytic M buffer (Sigma-Aldrich, Cat. #C2978), and 50 μg of total protein was loaded per lane. Protein concentrations were adjusted to ensure equal loading. Western blotting was performed using anti-IDH3A mouse monoclonal antibody (clone A-10) (Santa Cruz, Cat. #sc-398021) with a 400-fold dilution, and anti-GAPDH mouse monoclonal antibody (clone 1E6D9) (4,000-fold dilution, proteintech, 60004-1-Ig) as primary antibodies, diluted in 5% skim milk in Tween TBS. Horseradish peroxidase-conjugated anti-mouse IgG secondary antibodies (5,000-fold dilution in 1% skim milk in Tween-TBS; Cytiva Cat. #NA931) were used. Signals were detected using the ECL Plus Western Blotting Detection System (Amersham Cytiva, Cat. #RPN2232) according to the manufacturer's instructions. Chemiluminescent signals were acquired using the Amersham Imager 600 (Amersham Cytiva).

### Luciferase-based proliferation assays of cancer cells *in vitro*

MC38 cells were transiently transfected with pCDH/EF1α-Luc, which constitutively expresses luciferase, and cultured overnight. For direct co-culture assays, the resulting cells (MC38/EF1α-Fluc) and *Idh3a* KO tail-tip fibroblasts reconstituted with the IDH3α expression vector or empty vector were each seeded at 1.77 × 10^4^ cells/well in 24-well plates and co-cultured for 28 h. For conditioned medium assays, MC38/EF1α-Fluc cells (1.33 × 10^4^ cells/well in 24-well plates) were cultured for 28 h in conditioned media derived from the corresponding fibroblast populations. To evaluate the effect of fibroblast *Idh3a* status on cancer cell growth based on absolute bioluminescence intensity, cell lysates were collected in Passive Lysis Buffer (Promega, Cat. #E194A) and subjected to the luciferase assay (Promega, Cat. #E1910), according to the manufacturer’s instructions. Relative luciferase units (RLU) in each condition were calculated by normalizing luciferase activity to that obtained from MC38 cells processed under the same experimental conditions.

### Conditioned medium transfer assay

Immortalized *Idh3a* KO fibroblasts reconstituted with the pCDH/EF1α-mIDH3A-myc-His A or EV were seeded at 8 × 10^5^ cells per 10 cm φ dish in DMEM supplemented with 10% FBS and 1% penicillin-streptomycin (10S-DMEM) and incubated for 40 h. The resulting culture medium was then harvested and centrifuged at 10,000 rpm for 5 min to remove cellular debris. The clarified conditioned medium was mixed at a 1:1 ratio with fresh 10S-DMEM, and MC38 cells transiently transfected with pCDH/EF1α-Luc, which constitutively expresses luciferase, were cultured in this mixture (1.33 × 10^4^ cells per well in 24-well plates). Following a 28-h incubation, consistent with the co-culture assay, proliferation of MC38/EF1α-Fluc was assessed by measuring luciferase bioluminescence, according to the manufacturer’s instructions (Promega Cat. #E1483). Relative luciferase units (RLU) in each condition were calculated by normalizing luciferase activity to that obtained from MC38 cells processed under the same experimental conditions.

### Allografted tumor growth assay

MC38 cells (5 × 10^6^ cells/mL) or B16 cells (2.5 × 10^6^ cells/mL) were suspended in PBS and 100 μL of each cell suspension was transplanted intradermally into the dorsal flank of mice under anesthesia with 2.5% isoflurane gas in oxygen flow (1.5 L/min, Xenogen XGI-8). Tumor size was measured daily using digital calipers, and tumor volume was calculated as (length × width^2^)/2. Mice were monitored until reaching humane endpoints (maximum tumor volume 2,000 mm^3^ or severe ulceration) and euthanized by cervical dislocation. Animals were allocated to experimental groups based on predefined genetic background and treatment conditions (*e.g.*, genotype and tamoxifen administration), rather than by random assignment. Blinding was not applied during the experiments, as group allocation was defined by genotype and treatment, and all primary outcomes were assessed using objective quantitative measurements. Group sizes for mouse experiments were determined based on prior experience and published studies in the field, aiming to ensure reproducibility and detect biologically meaningful effects. No formal statistical power calculation was performed.

### Allografted tumor growth assay after bone marrow-specific *Idh3a* KO

Bone marrow-derived cells (BMDCs) were collected from the tibias and femurs of 4-6-week-old female *Idh3a*^fl/fl^; CreER^T2+/+^; C57BL/6-Tg(CAG-EGFP) or C57BL/6-Tg(CAG-EGFP) donor mice. Cells were resuspended and filtered in PBS, and 1 × 10^7^ BMDCs in 200 μL PBS were injected intravenously into recipient C57BL/6 N mice (Japan SLC, Inc.). Gender-matched recipient mice (8-9-week-old) received whole-body γ-irradiation at a dose of 9.5 Gy using the Cs-137 source of Gamma cell 40 Exactor (MDS Nordion) to ablate endogenous bone marrow and hematopoietic progenitor cells prior to transplantation. Thirty days later, 100 mg/kg of tamoxifen was administered for 5 consecutive days (as described above). Seven days after the last tamoxifen injection, mice were intradermally transplanted with MC38 cells in PBS (as described above), and tumor growth was monitored.

### FACS analysis

To evaluate bone marrow repopulation efficiency following whole body γ-irradiation and bone marrow transplantation, peripheral blood samples were collected by facial vein puncture into EDTA-containing anti-coagulant tubes. Red blood cells were subsequently lysed in Tris-buffered ammonium chloride solution (1 part 0.17 M Tris(hydroxymethyl)aminomethane-HCl and 9 parts 0.16 M NH_4_Cl). The remaining leukocytes were incubated with TruStain FcX^TM^ (anti-mouse CD16/32) antibody (Biolegend, Cat. #101320) for Fc blocking, followed by staining with PE/Cyanine7 anti-mouse CD45 antibody (clone 30-F11) (Biolegend, Cat. #103114), according to the manufacturer’s instructions. Viable donor-derived GFP^+^ leukocytes were identified as 7-amino-actinomycin D (7AAD)-negative cells (BD Pharmingen, Cat. # 51-2359KC). For tumor infiltration analysis, tumors were surgically excised from euthanized mice, digested with Collagenase Type IV (Gibco, Cat. #17104019) and passed through 100-μm mesh filters to obtain single-cell suspensions. The isolated cells were stained as described above to identify the CD45^+^ populations within viable cell fractions. All samples were analyzed using BD FACSCanto II flow cytometer.

### Single cell RNA seq analysis

The scRNA-seq dataset from patients with triple-negative breast cancer (TNBC) was downloaded from the Gene Expression Omnibus (GEO) database (accession number: GSE246613). The processing and classification of immune cells (B cells, T cells, mast cells, and myeloid cells) and non-immune cells (endothelial cells, epithelial cells, fibroblasts, and perivascular-like cells [PVL]) were performed according to previously described methods [15]. Gene expression analyses were performed using Scanpy (version 1.10.4) and NumPy (version 1.24.4). Uniform Manifold Approximation and Projection (UMAP) visualization was generated using Python (version 3.11.6). Only samples derived from patients diagnosed with triple-negative breast cancer (TNBC) that met the quality control criteria defined in the original GEO dataset (GSE246613) were included in the analysis. No additional inclusion or exclusion criteria were applied by the authors. Because this study exclusively analyzed publicly available scRNA-seq data from the GEO database, there was no attrition or dropout of subjects during data collection or analysis. All samples analyzed in this study were derived from patients diagnosed with triple-negative breast cancer (TNBC), which predominantly affects female patients; sex information was obtained from the original GEO dataset metadata, and no additional stratification by sex/gender was performed. No group allocation was performed, as all analyses were conducted on publicly available datasets without experimental intervention. Blinding was not applicable because the study involved secondary analysis of publicly available datasets without experimental intervention. Sample size was determined by the publicly available dataset (GSE246613), and no a priori power calculation was applicable.

### Immunofluorescence analysis

Excised tumors were embedded in OCT compound (Sakura Finetech, Cat. # 4583). Frozen tumor sections were fixed in ice-cold 4% PFA for 15 min. Samples were blocked with Protein Block, Serum-Free (Dako, Cat. #X0909) and incubated overnight at 4°C with anti-αSMA Alexa 647-conjugated antibody (clone 1A4) (BioLegend, Cat. #614855; 1:100 dilution in Protein Block, Serum-Free) and Purified Rat anti-Mouse CD31 antibody (clone MEC 13.3) (BD Pharmingen, Cat. #550274; 1:50 dilution in Protein Block, Serum-Free). The following day, samples were washed and stained with goat anti-rat Alexa Fluor 546 antibody (Invitrogen, RRID: Cat. #AB_10561522; 1:1000 dilution in Protein Block, Serum-Free to detect CD31) at room temperature for 1 h. Samples were washed with Tween Tris-buffered saline and mounted with ProLong Glass Antifade Mountant with NucBlue Stain (Invitrogen, Cat. #P36981). Stained slides were imaged using Olympus Fluoview FV10i confocal laser scanning microscope, and fluorescence signals were analyzed using ImageJ (Fiji).

### Quantitative real-time PCR (qRT-PCR)

C57BL/6 (B6), conventional *Idh3a* heterozygous KO (WT/KO), and *Idh3a*^fl/fl^; CreER^T2+/+^ (cKO) mice were treated with tamoxifen for 5 consecutive days (100 mg/kg/day). Representative organs were surgically excised 20 days after the initial tamoxifen treatment. Total RNA was extracted from these tissues using Sepasol-RNA I Super G (Nacalai Tesque, Cat. #09379-84) treated with DNase I (Invitrogen, Cat. #10694233), and subjected to reverse transcription using the PrimeScript RT reagent Kit (Perfect Real Time) (Takara Bio, Cat. #RR037), according to the manufacturer’s instructions. Quantitative PCR was performed using TB Green Premix Ex Taq II (Tli RNaseH Plus) (Takara Bio, Cat. #RR820). PCR primers were designed to span the exon 5-6 junction of mouse *Idh3a*; Idh3a-e5-6_F primer: 5′-GAGAGAACACGGAAGGAGAATAC-3′ and Idh3a-e5-6_R primer: 5′-CGAAGGCAAACTCTGCAATG-3′, corresponding to the floxed genomic region. Gene expression levels were normalized to mouse β-actin quantified by quantitative PCR using primers; MA050368-F primer: 5′-CATCCGTAAAGACCTCTATGCCAAAC-3′ and MA050368-R primer: 5′-ATGGAGCCACCGATCCACA-3′.

### Code and protocol availability

No custom code was developed for this study. All data processing and analyses were conducted using publicly available software, including Scanpy, NumPy, and Python, as described above. Full step-by-step protocols were not deposited in external protocol repositories (*e.g.*, protocols.io); all relevant procedures are described in the Materials and Methods.

### Ethics

All mouse experiments were approved by the Kyoto University Animal Experiment Committee, and mice were euthanized when the total tumor volume reached 2,000 mm³ or when severe tamoxifen-induced toxicity was observed. All human-related analyses in this study were performed exclusively using publicly available single-cell RNA sequencing datasets obtained from the Gene Expression Omnibus (GEO) database (accession number: GSE246613). No human specimens were newly collected, and no analyses involving identifiable individual-level human data were conducted by the authors. Therefore, approval from an institutional ethics committee was not required.

## Results

### Generation of conventional and tamoxifen-inducible conditional *Idh3a* KO mice

**Fig. 1:**
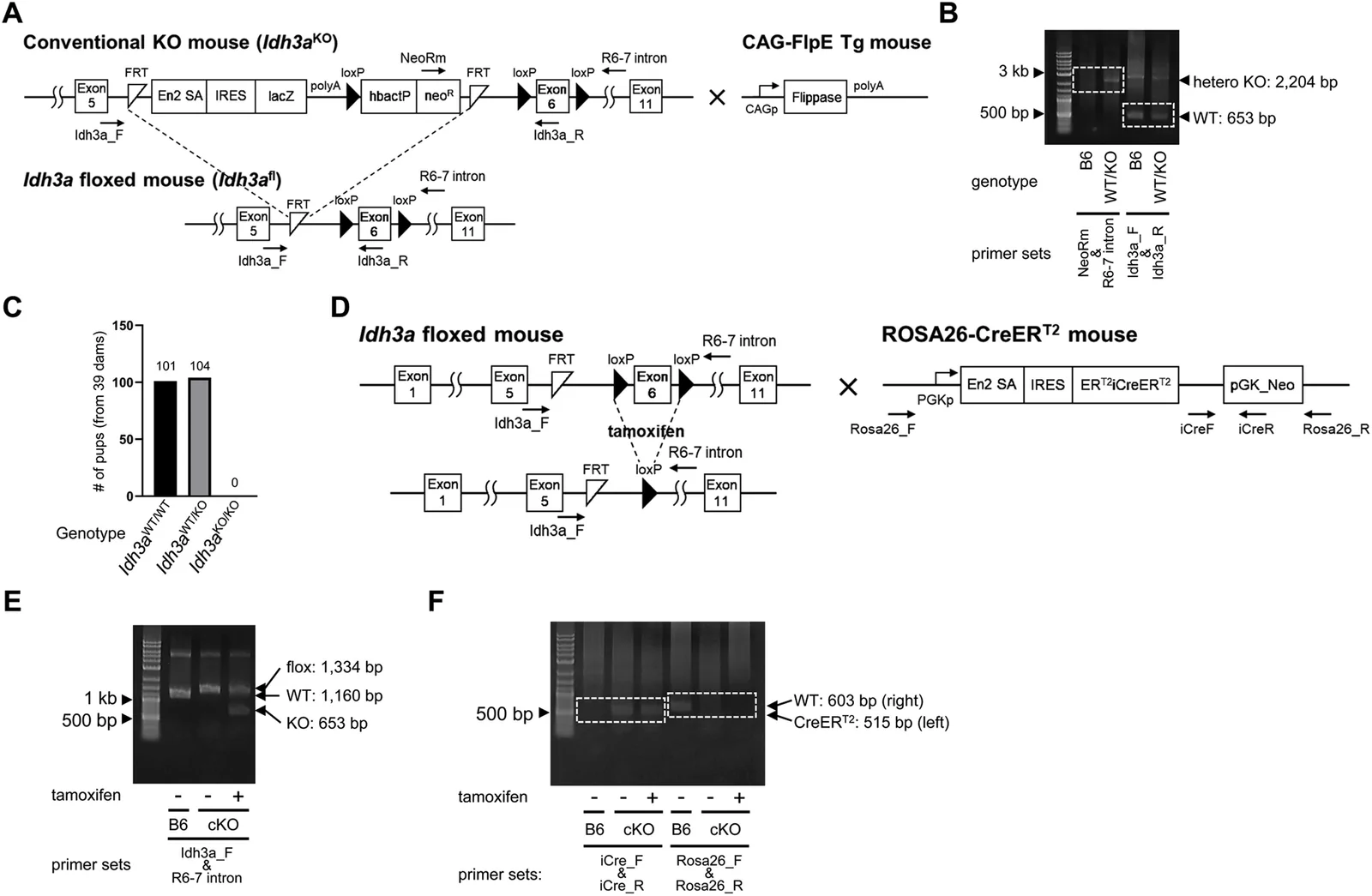
**Generation of conventional and tamoxifen-inducible conditional *Idh3a* KO mice. A**. Schematic representation of the conventional *Idh3a* KO allele (*Idh3a*^KO^, upper left), *Idh3a* floxed allele (*Idh3a*^fl^, lower left), and CAG-FlpE gene (upper right). The conventional KO allele contains a poly A sequence downstream of exon 5 to terminate transcription. FRT: flippase recognition target, en2SA: En2 splice acceptor site, IRES: internal ribosome entry site, lacZ: β-galactosidase gene, hbactP: human beta-actin promoter, neoR: neomycin resistance gene, poly A: polyadenylation signal, and loxP: bacteriophage P1-derived loxP site. **B**. Genotyping PCR of C57BL/6 (B6) and conventional *Idh3a* heterozygous KO (WT/KO) mice using NeoRm and R6-7 intron primers detects the knock-in cassette (left, 2,204 bp), whereas PCR using Idh3a_F and Idh3a_R primers amplifies the wild-type allele (right, 653 bp). **C**. Genotype-specific total offspring obtained from 39 pairs of *Idh3a* heterozygous KO intercrosses. **D**. Schematic representation of the *Idh3a* floxed allele (upper left), the ER^T2^iCreER^T2^ expression cassette in ROSA26-CreER^T2^ mice (upper right), and the resulting *Idh3a* KO allele generated after crossing *Idh3a* floxed mice with ROSA26-CreER^T2^ mice followed by tamoxifen-induced Cre-mediated recombination. PGKp: phosphoglycerate kinase 1 promoter. **E**. PCR genotyping of the *Idh3a* locus in C57BL/6 (B6) and *Idh3a*^fl/fl^; CreER^T2+/+^ (cKO) mice with or without tamoxifen treatment was performed using Idh3a_F and R6-7-intron primers, yielding PCR products of 1,160 bp (WT), 1,334 bp (floxed), and 653 bp (KO). **F**. PCR genotyping of the ROSA26-Cre-ER^T2^ allele. The CreER^T2^ transgene is detected using iCreF and iCreR primers (515 bp), whereas the wild-type ROSA26 allele is detected using Rosa26_F and Rosa26_R primers (603 bp).

To investigate the role of stromal cell mitochondria in tumor growth, we first generated conventional *Idh3a* knockout (KO) mice. *Idh3a* encodes the α subunit (IDH3α) of the heterotetrametric isocitrate dehydrogenase 3 (IDH3), which catalyzes a key rate-limiting step in the TCA cycle. In the murine *Idh3a* gene, Tyr153 and Lys200, critical residues for catalytic activity, are encoded by exons 5 and 6, respectively (UniProt accession Q9D6R2). Therefore, to disrupt IDH3α function, the knockout construct was designed to target exon 6, thereby preventing expression of the catalytically active enzyme. The targeting vector was obtained from the European Conditional Mouse Mutagenesis (EUCOMM) program and microinjected into C57BL/6-derived embryonic stem cells to generate conventional *Idh3a* KO mice (*Idh3a*^KO^ herein; Fig. 1A, upper left). Heterozygous founders carrying the expected transgene were identified by PCR genotyping (Fig. 1B); PCR using Idh3a_F and Idh3a_R primers produced a 653 bp product from the wild-type allele in both parental C57BL/6 and heterozygous *Idh3a*^WT/KO^ mice (Fig. 1B, right), whereas NeoRm and R6-7 intron primers yielded a 2,204 bp band only in transgene-positive mice (Fig. 1B, left). Intercrosses of heterozygous *Idh3a* KO mice (*Idh3a*^WT/KO^) yielded viable *Idh3a*^WT/WT^ and *Idh3a*^WT/KO^ offspring, whereas no homozygous *Idh3a*^KO/KO^ mice were obtained (Fig. 1C), indicating that complete loss of *Idh3a* results in embryonic lethality, as reported previously [16].

To circumvent this lethality, we next established a tamoxifen-inducible *Idh3a* conditional KO (cKO) mouse line. The FRT-flanked DNA fragment in the original targeting vector, containing a polyadenylation (poly A) signal, neomycin-resistant (neo^R^) cassette, and associated sequences, was excised by crossing *Idh3a*^WT/KO^ mice with CAG-FlpE transgenic mice (C57BL/6-Tg(CAG-flpe)36Ito/ItoRbrc, Riken Bio Resource Center; Fig. 1A, upper right), resulting in loxP sites flanking exon 6 (*Idh3a*^fl^ herein; Fig. 1A, lower left). Homozygous *Idh3a*^fl/fl^ mice were then crossed with B6.ROSA26Tm(SAEiCE)16Card, RIKEN BRC (ROSA26-CreER^T2^ mice, herein) to create tamoxifen-inducible *Idh3a* cKO mice (*Idh3a*^fl/fl^; CreER^T2+/+^; Fig. 1D). Genotyping of the established line in the absence of tamoxifen using the Idh3a_F and R6-7 intron primers confirmed the presence of a 1,334 bp product from the floxed allele, but not the shorter 1,160 bp product from the wild-type allele, indicating the homozygosity of the floxed allele (Fig. 1E). Homozygosity of the expression cassette for the ER^T2^iCreER^T2^ (hereafter referred to as CreER^T2^) at the ROSA26 safe-harbor locus in the established mice was verified by two PCR-based genotyping assays; detection of a 515 bp product using the iCreF and iCreR primers (Fig. 1F, left), and absence of a 603 bp product corresponding to the wild-type allele using the Rosa26_F and Rosa26_R primers (Fig. 1F, right). Following intraperitoneal administration of tamoxifen, genotyping analysis of *Idh3a*^fl/fl^; CreER^T2+/+^mice revealed the appearance of a recombined shorter PCR product (653 bp) in addition to the floxed band (1,334 bp), indicating successful excision of exon 6 and functional inactivation of *Idh3a* upon tamoxifen treatment (Fig. 1E).

### Reduced *Idh3a* expression in the conventional heterozygous KO and tamoxifen-inducible cKO mouse models

**Fig. 2:**
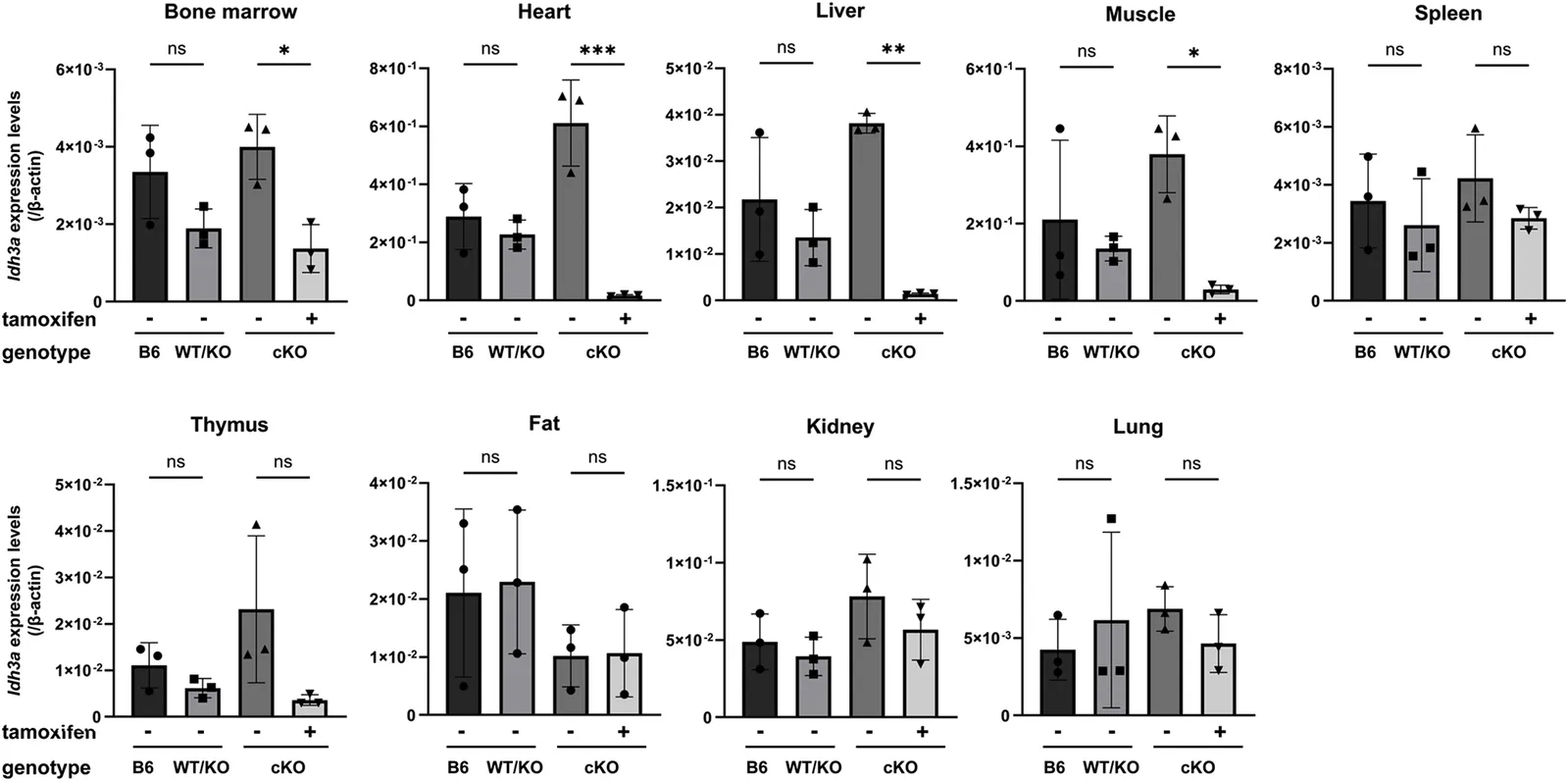
**Reduced *Idh3a* expression in the conventional heterozygous KO and tamoxifen-inducible cKO mouse models.***Idh3a* mRNA expression levels quantified by qRT-PCR in the indicated organs from female C57BL/6 (B6), conventional *Idh3a* heterozygous KO (WT/KO), and *Idh3a*^fl/fl^; CreER^T2+/+^ (cKO) mice with or without tamoxifen administration. Data represent mean ± SD (n = 3 mice per group). Statistical significance was determined by one-way ANOVA (*P < 0.05; **P < 0.01; ***P < 0.001; ns, not significant).

We evaluated the influence of *Idh3a* knockout achieved by two distinct genetic systems, the conventional KO (Fig. 1A, upper left) and the tamoxifen-inducible cKO (Fig. 1D, lower left), on intact *Idh3a* expression levels. Because the qRT-PCR experiments were performed using primers annealing to the flanking region of exons 5 and 6, no amplification is theoretically possible when the floxed exon 6 encoding the catalytic region is deleted. Total RNA was extracted from multiple major organs, all of which are known to be heavily dependent on ATP production and utilization [[17], [18], [19]], and was subjected to subsequent analyses. Because conventional homozygous *Idh3a* KO mice exhibited embryonic lethality (Fig. 1C), we first examined *Idh3a* mRNA expression levels in the conventional heterozygous KO line (*Idh3a*^WT/KO^). qRT-PCR analysis showed a modest, though not statistically significant, reduction in intact *Idh3a* transcript levels compared with C57BL/6 mice (*Idh3a*^WT/WT^), consistent with monoallelic disruption of the gene (Fig. 2, left side of each graph).

We next assessed the KO efficiency in the tamoxifen-inducible cKO model (*Idh3a*^fl/fl^; CreER^T2+/+^), which allowed efficient postnatal gene disruption upon Cre-mediated recombination. cKO mice were administered tamoxifen intraperitoneally at a dose of 100 mg/kg for 5 consecutive days and sacrificed on day 20 after the start of tamoxifen administration. A marked reduction in the intact *Idh3a* mRNA levels was observed across most tissues examined compared with vehicle-treated controls (Fig. 2, right side of each graph). The reduction of intact *Idh3a* transcript levels was particularly pronounced in metabolically active tissues, such as bone marrow, heart, liver, and skeletal muscle, all of which depend heavily on mitochondrial ATP generation for their function [[17], [18], [19]]. These data confirm that tamoxifen administration effectively induces recombination of the floxed *Idh3a* allele, producing a systemic and functional loss of *Idh3a* expression. This inducible cKO system provides a more suitable model than the conventional heterozygous KO mouse line for investigating the impact of stromal TCA cycle-dependent mitochondrial function on tumor progression.

### Host *Idh3a* deficiency promotes tumor growth in a cancer cell type-dependent manner

**Fig. 3:**
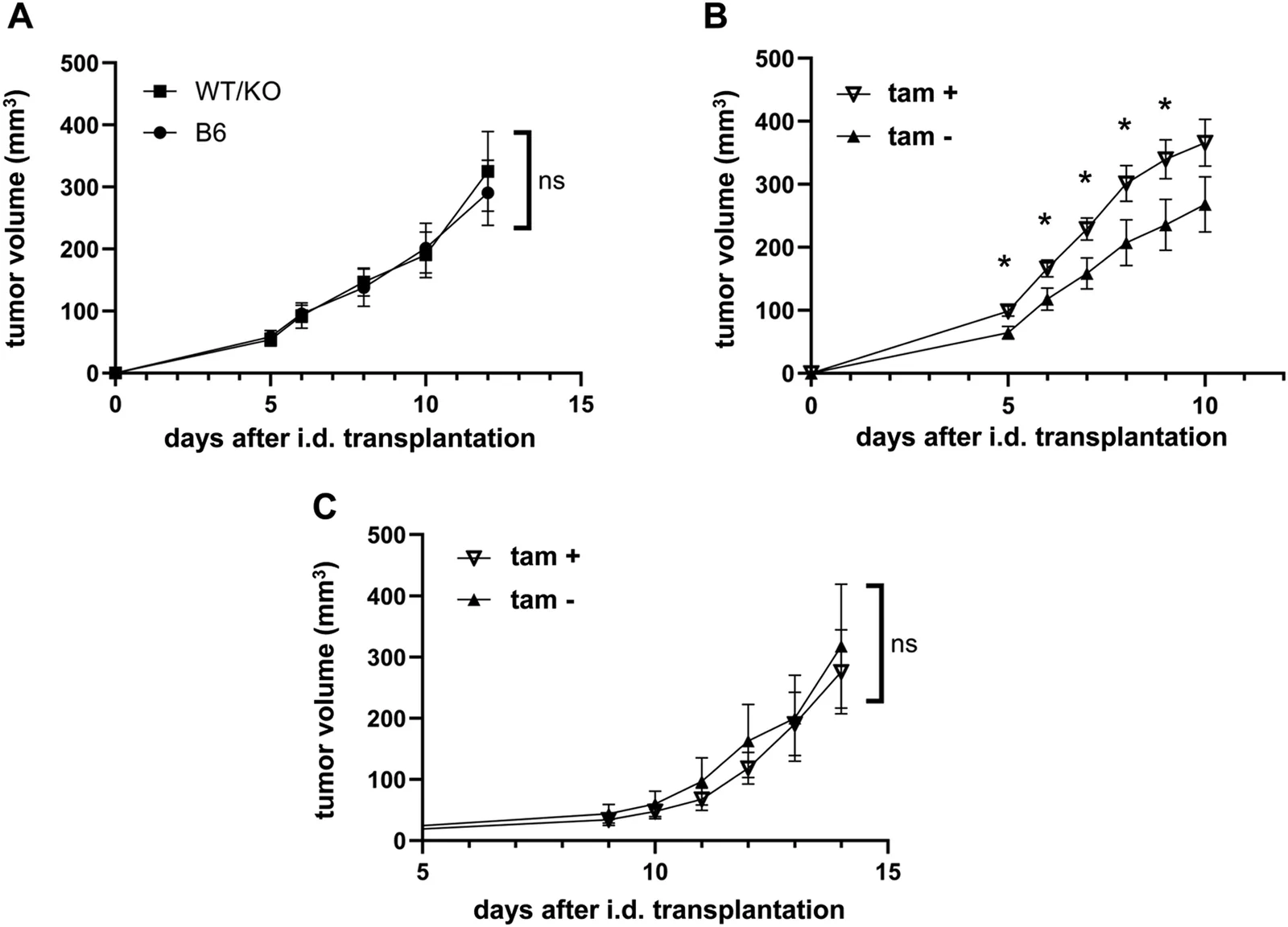
**Host *Idh3a* deficiency promotes tumor growth in a cancer cell type-dependent manner. A**. Growth curves of tumors following intradermal transplantation of MC38 cells (5 × 10^5^ cells/mouse) into conventional *Idh3a* heterozygous KO (WT/KO) and C57BL/6 (B6) mice (n = 16 per genotype). **B,C**. Growth curves of tumors following intradermal transplantation of MC38 cells (**B**, 5 × 10^5^ cells/mouse) or B16 cells (**C**, 2.5 × 10^5^ cells/mouse) into *Idh3a*^fl/fl^; CreER^T2+/+^ mice one week after the final injection of corn oil (tam-) or tamoxifen (tam+) (100 mg/kg/day for 5 consecutive days) (B: n = 27 in tam- and n = 30 in tam+, **C**: n = 14 per genotype). Data are presented as mean ± SEM. Statistical significance was analyzed using Student’s t-test (*P < 0.05; ns, not significant).

The expression levels of intact *Idh3a* were moderately reduced in the conventional heterozygous KO mice and further diminished in the tamoxifen-inducible cKO mice following Cre-mediated recombination (Fig. 2). To evaluate the functional impact of host *Idh3a* deficiency on tumor growth, we first intradermally transplanted MC38 murine colon adenocarcinoma cells endogenously expressing functional IDH3α into the dorsal flanks of conventional heterozygous KO mice (*Idh3a*^WT/KO^) or C57BL/6 mice (*Idh3a*^WT/WT^). Tumor growth was monitored daily; however, no significant difference was observed between mice with different *Idh3a* status (Fig. 3A). Given that expression levels of intact *Idh3a* were only partially decreased in the conventional KO model (Fig. 2), we next employed the tamoxifen-inducible cKO mice to achieve more efficient loss of functional IDH3α. The cKO mice (*Idh3a*^fl/fl^; CreER^T2+/+^) were injected intraperitoneally with tamoxifen (100 mg/kg/day) for 5 consecutive days and intradermally transplanted with MC38 cell suspension on day 11 after the start of tamoxifen administration. The tumor growth assay clearly demonstrated that tumors in the *Idh3a* KO group grew significantly faster than those in the intact *Idh3a* (vehicle-treated control) group (Fig. 3B). This result indicates that host-derived IDH3α exerts a tumor-suppressive influence *via* non-malignant stromal cells within the tumor microenvironment. To test whether this tumor-suppressive effect of host IDH3α is generalizable across different cancer types, we used another syngeneic cell line, B16 murine melanoma. In this setting, *Idh3a* deficiency in the host did not significantly affect tumor growth, suggesting that the tumor-suppressive function of host IDH3α may depend on the tumor cell type or microenvironmental context (Fig. 3C).

### Identification of *IDH3A*-expressing cell populations by single-cell RNA sequencing

**Fig. 4:**
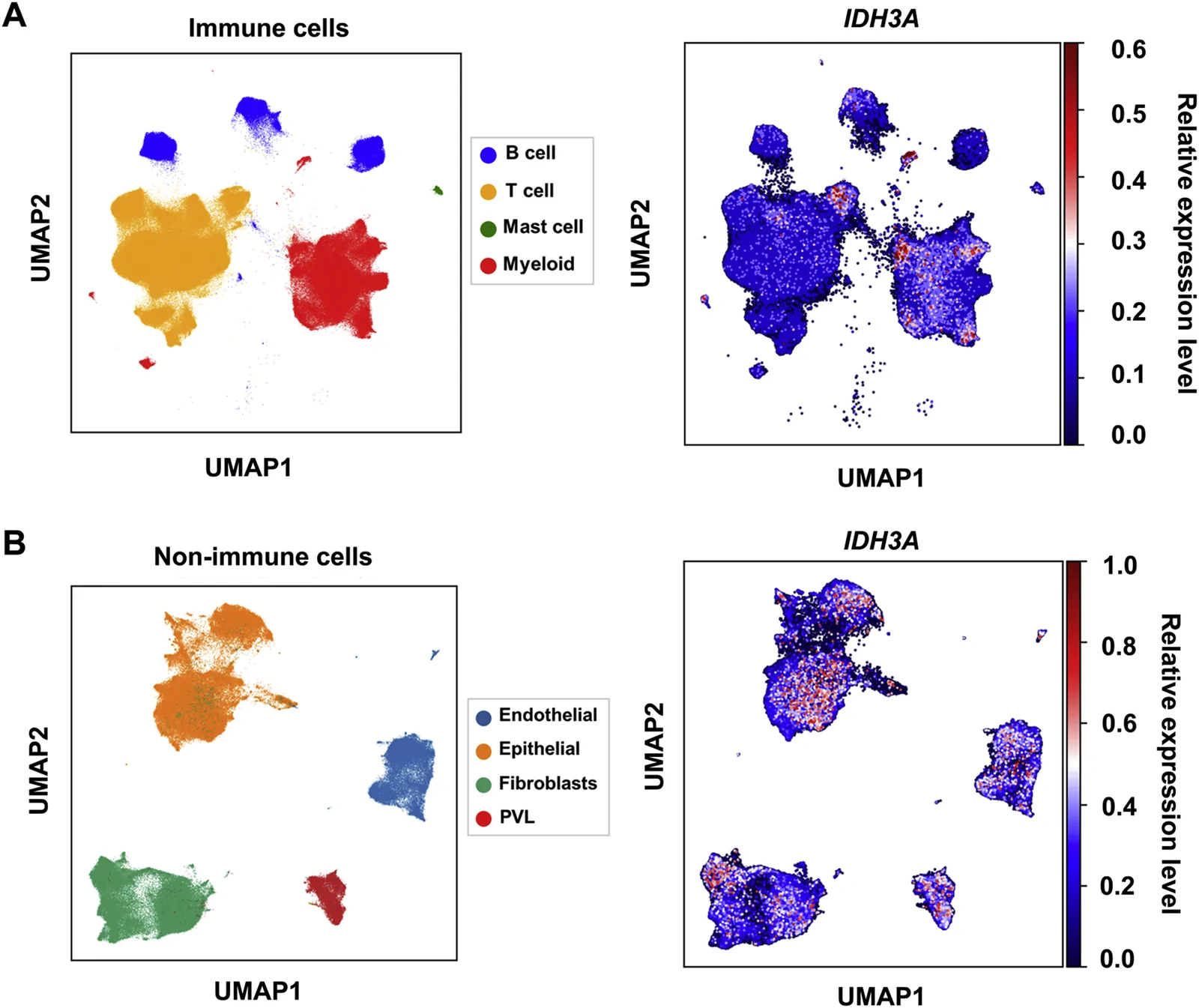
**Identification of IDH3A-expressing cell populations by Single-cell RNA Sequencing. A,B**. UMAP projections of immune (**A**) and non-immune (**B**) cells from TNBC clinical samples, colored by the indicated cell lineages (left in each panel) and overlaid with *IDH3A* expression levels (right in each panel).

To identify cell lineages potentially responsible for the tumor-suppressive effects of host IDH3α, we analyzed publicly available single-cell RNA sequencing datasets from human tumor specimens. This approach enabled an unbiased assessment of *IDH3A* mRNA distribution across diverse cell populations within the tumor microenvironment (Fig. 4). Cells were broadly classified into immune and non-immune compartments (Fig. 4A and 4B, respectively). Among immune cell populations, IDH3A expression was detectable across multiple lineages, with transcripts observed in both lymphoid and myeloid compartments (Fig. 4A). Within the non-immune compartment, *IDH3A* transcripts were distributed among endothelial, epithelial, fibroblastic, and perivascular-like stromal cells, although no single subcluster exhibited uniquely dominant expression compared with the others (Fig. 4B). These results demonstrate that *IDH3A* is expressed across multiple cell types within the tumor microenvironment, encompassing both immune and non-immune lineages.

### Bone marrow-derived cells are not responsible for IDH3α-dependent tumor growth suppression

**Fig. 5:**
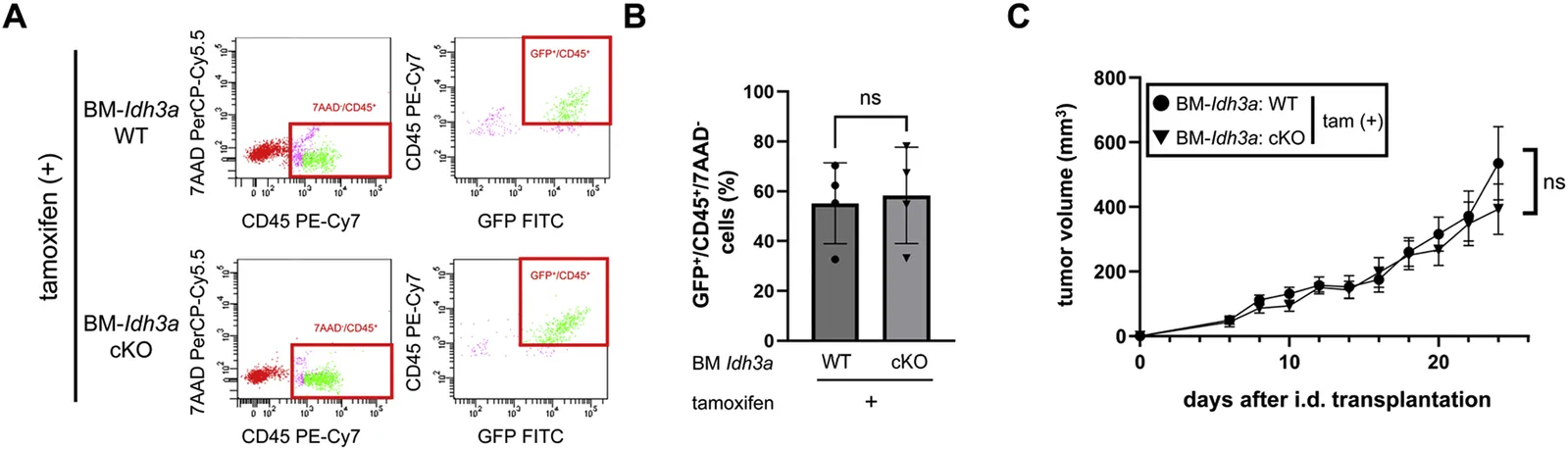
**Bone marrow-derived cells are not responsible for IDH3α-dependent tumor growth suppression. A,B**. Flow cytometry analyses for viability (7AAD), hematopoietic cells (CD45), and GFP (transplanted BMDC) (**A**) and quantification of donor-derived tumor-infiltrating CD45^+^/GFP^+^/7AAD^−^ cells (**B**) in wild-type recipient mice. Tumors were harvested 24 days after MC38 allograft transplantation. **C**. Growth curves of MC38 tumors in bone marrow-reconstituted wild-type female recipient mice with or without tamoxifen treatment, following tamoxifen-induced knockout of *Idh3a* in bone marrow-derived cells. Data are presented as mean ± SD (n = 4 in **B**) and SEM (n = 9 in **C**). Statistical significance was analyzed using Student’s t-test (ns, not significant).

To determine whether the tumor-suppressive effect observed in the *Idh3a* cKO mice was mediated by hematopoietic cells, we performed bone marrow transplantation experiments. Wild-type recipient mice were lethally γ-irradiated with a total dose of 9.5 Gy and reconstituted with bone marrow cells harvested from either *Idh3a* wild-type donor mice (C57BL/6-Tg(CAG-EGFP)) or *Idh3a* cKO donor mice (*Idh3a*^fl/fl^; CreER^T2+/+^; C57BL/6-Tg(CAG-EGFP)), both of which constitutively expressed EGFP for lineage tracing. One month later, flow cytometric analysis confirmed that >90% of circulating CD45^+^ hematopoietic cells exhibited EGFP fluorescence, indicating successful engraftment of donor-derived bone marrow cells (Supplementary Fig. 2). To induce *Idh3a* knockout specifically in bone marrow-derived cells, the chimeric mice received daily intraperitoneal injections of tamoxifen (100 mg/kg/day) for 5 consecutive days, followed by a 1-week recovery period before intradermal transplantation of MC38 tumor cells. Tumor growth was monitored daily, and tumors were analyzed at the endpoint for infiltration of donor-derived immune cells based on CD45 and EGFP expression (Fig. 5A, B). Flow cytometric quantification showed comparable levels of EGFP^+^ CD45^+^ tumor-infiltrating hematopoietic cells in both groups (Fig. 5B). No significant difference in tumor growth was observed between mice harboring wild-type or *Idh3a*-deficient bone marrow (Fig. 5C, Supplementary Fig. 3). These data demonstrate that loss of *Idh3a* in hematopoietic cell lineages does not account for the tumor-suppressive phenotype observed in the tumor-bearing *Idh3a* cKO mice.

### Fibroblast IDH3α suppresses tumor cell proliferation *via* a contact-dependent mechanism

**Fig. 6:**
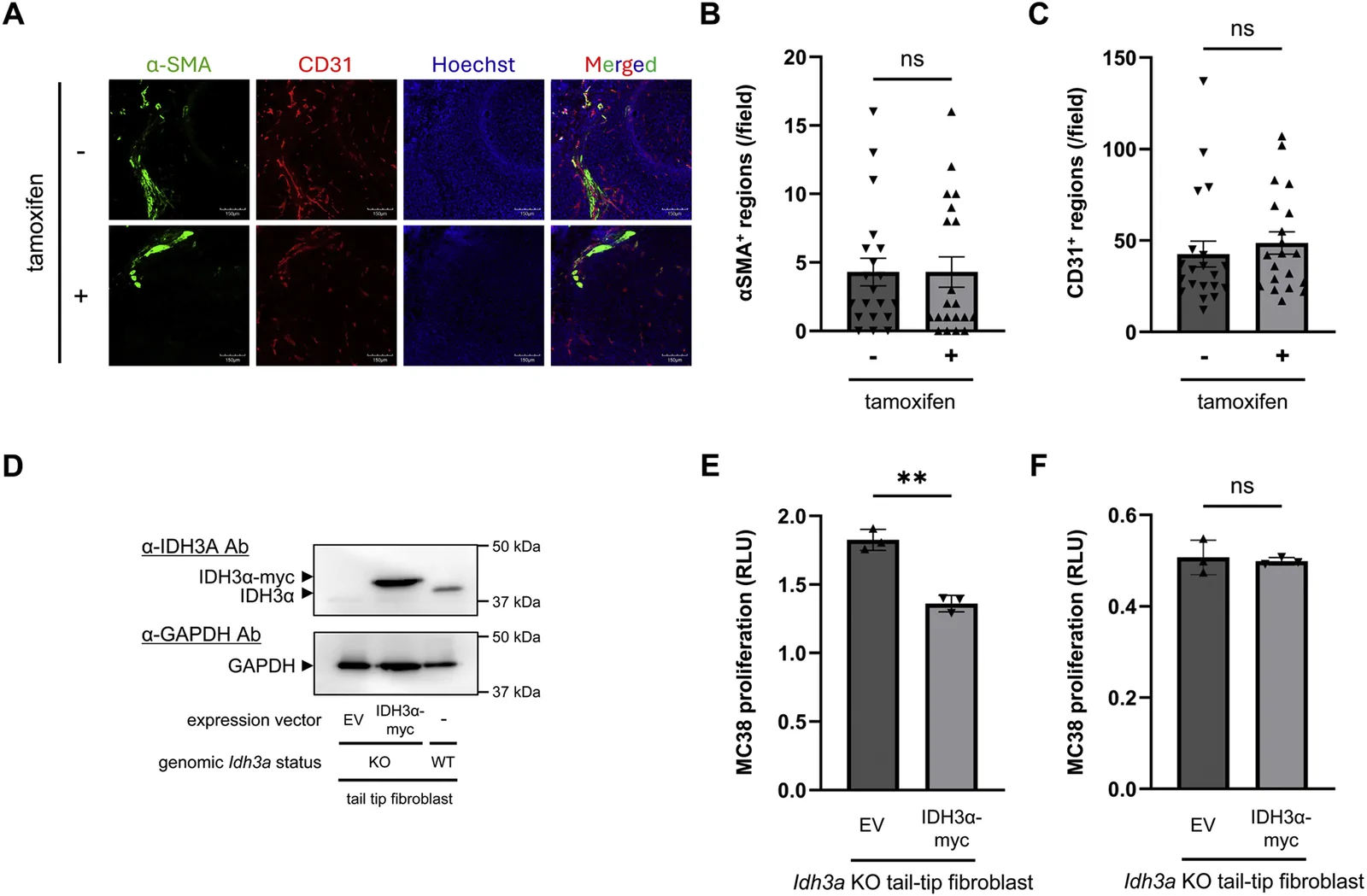
**Fibroblast IDH3α suppresses tumor cell proliferation *via* a contact-dependent mechanism. A**-**C**. Immunofluorescence analysis of MC38 tumors from *Idh3a*^fl/fl^; CreER^T2+/+^ (cKO) mice treated with or without tamoxifen. Representative confocal images showing α-smooth muscle actin (α-SMA; green), CD31 (red), and nuclei (Hoechst33342; blue) (**A**), and quantification of α-SMA-positive stromal regions (**B**) and CD31-positive microvessel density (**C**). Quantification was performed using 4 independent tumors per group with 5 randomly selected fields per tumor. **D**. Western blot analyses using the indicated antibodies in *Idh3a*-KO (KO) and *Idh3a*-intact (WT) tail-tip fibroblasts reconstituted with empty vector (EV), IDH3α-expression vector (IDH3α-myc), or left untreated (-). **E,F**. Luciferase-based proliferation assays of MC38/EF1α-Fluc cells performed either in direct co-culture with *Idh3a*-KO tail-tip fibroblasts reconstituted with an IDH3α expression vector (IDH3α-myc) or empty vector (EV) (**E**), or in conditioned media derived from the corresponding fibroblast populations (**F**). n = 3. Data in **B, C, E**, and **F** are shown as mean ± SD. Statistical significance was analyzed using Student’s t-test (**P < 0.01; ns, not significant).

The results in Fig. 5 suggest that tissue-resident stromal cell populations with limited migratory capacity, which are not replaced by bone marrow transplantation and constitute radioresistant stromal compartments, such as fibroblasts, may mediate IDH3α-dependent regulation of tumor growth. To first determine whether *Idh3a* status affects the recruitment of fibroblasts into tumors, we examined fibroblast infiltration in tumor tissues. Tumors were generated by transplanting *Idh3a* wild-type MC38 cells into *Idh3a* wild-type and *Idh3a* cKO mice, followed by immunofluorescence staining using an antibody against α-SMA, a representative fibroblast marker. The number of α-SMA-positive fibroblasts infiltrating the tumor tissues was comparable between the two groups, indicating that fibroblast infiltration into tumors was not influenced by recipient *Idh3a* status (Fig. 6A–C).

Having established that fibroblast abundance within tumors was not affected by *Idh3a* status, we next analyzed the impact of fibroblast *Idh3a* status on cancer cell proliferation *in vitro*. MC38/EF1α-Fluc stable transfectants, which constitutively express luciferase, were co-cultured with equal numbers of tail-tip fibroblasts harboring different *Idh3a* genotypes (Fig. 6D, Supplementary Fig. 1), and cancer cell growth was monitored by changes in luciferase activity. Proliferation of cancer cells was significantly suppressed when co-cultured with fibroblasts expressing IDH3α, but not with *Idh3a* KO fibroblasts (Fig. 6E).

To further assess whether this fibroblast-mediated tumor-suppressive effect was dependent on soluble factors, MC38/EF1α-Fluc cells were cultured with conditioned media derived from fibroblasts differing in *Idh3a* status. Under these conditions, no significant change in cancer cell proliferation was observed, indicating that the IDH3α-dependent tumor-suppressive effect of fibroblasts is not mediated by secreted factors but instead requires direct cell-cell interactions (Fig. 6F).

## Discussion

In our previous studies, we reported that, in cancer cells aberrantly overexpressing IDH3α, IDH3 does not primarily function in its canonical oxidative decarboxylation reaction converting isocitrate to α-ketoglutarate within the TCA cycle [20]. Instead, IDH3 promotes a reductive carboxylation reaction that converts α-ketoglutarate to isocitrate, leading to a marked reduction in intracellular α-ketoglutarate levels [20]. This depletion of α-ketoglutarate results in activation of HIF-1 signaling and thereby drives malignant progression of cancer cells [20]. Although these findings have begun to suggest the role of IDH3 in cancer cells, the impact of IDH3 in non-cancerous cells within the tumor microenvironment on tumor growth has remained largely unclear. Under these circumstances, in the present study, we employed a tumor transplantation model using conditional *Idh3a* knockout mice in which systemic deletion of *Idh3a* can be induced in a tamoxifen-dependent manner. Using this model, we found that knockout of *Idh3a* in non-cancerous cells infiltrating tumors significantly accelerates tumor growth. Furthermore, our results indicate that fibroblasts, rather than bone marrow-derived cells, are an important stromal cell type contributing to IDH3α-dependent tumor growth suppression. Notably, our *in vitro* data suggest that this tumor-suppressive effect cannot be explained by soluble factors alone but instead requires direct interactions between fibroblasts and cancer cells.

In stromal fibroblasts, IDH3α has been reported to function as an important metabolic checkpoint. Zhang et al. demonstrated that IDH3α expression is reduced during the conversion of normal fibroblasts into cancer-associated fibroblasts (CAFs), resulting in a metabolic shift from oxidative phosphorylation toward aerobic glycolysis [21]. Mechanistically, TGF-β1- or PDGF-induced fibroblast activation was associated with miR-424-mediated downregulation of IDH3α, a reduced α-ketoglutarate-to-fumarate/succinate ratio, stabilization of HIF-1α, and increased glycolytic activity [21]. Importantly, IDH3α expression was decreased in CAFs isolated from human tumors, whereas ectopic expression of IDH3α suppressed CAF formation [21]. These observations raised the possibility that maintenance of IDH3α-dependent mitochondrial metabolism in stromal fibroblasts may restrain tumor progression *in vivo*.

Our present *in vivo* findings extend these previous observations. In a tumor transplantation model in which *Idh3a* was systemically deleted in the recipient mice but not in the transplanted cancer cells, transplantation of the murine colon carcinoma cell line, MC38, resulted in accelerated tumor growth, suggesting that IDH3α in non-malignant host cells may function in a tumor-suppressive manner. In contrast, no comparable effect of host *Idh3a* status was observed when the melanoma cell line, B16, was transplanted into the same cKO mice. However, because only one colorectal carcinoma cell line and one melanoma cell line were examined in the present study, it remains unclear whether this difference reflects tumor-type specificity or cell line-specific biological properties. Thus, the present findings should be interpreted as indicating that the influence of stromal IDH3α on tumor growth is context dependent rather than establishing a general difference between colorectal cancer and melanoma.

One potential factor underlying the different responses of MC38 and B16 tumors is their distinct immunogenicity [22,23]. MC38 tumors are generally considered highly immunogenic and tend to form so-called “hot tumors,” whereas B16 tumors are poorly immunogenic and are regarded as typical “cold tumors” that establish an immunosuppressive tumor microenvironment [24,25]. However, genetic deletion of *Idh3a* in hematopoietic stem cells did not recapitulate the accelerated tumor growth observed in the systemic knockout setting, indicating that hematopoietic lineages are unlikely to account directly for this phenotype. In contrast, *in vitro* co-culture experiments suggested a functional contribution of fibroblasts. These findings raise the possibility that the differential effects observed in MC38 and B16 tumors may reflect context-dependent interactions between IDH3α-expressing stromal cells, such as fibroblasts, and tumor microenvironments with distinct immunological properties. Together, these observations suggest that the tumor-suppressive function of host IDH3α may be mediated predominantly by non-hematopoietic stromal cells, such as fibroblasts. The mechanisms underlying the differential effects observed in MC38 and B16 tumors remain to be determined.

In this study, single-cell RNA sequencing analysis of human tumor specimens revealed that *IDH3A* expression was detected across multiple cell lineages. Within immune cell populations, IDH3A transcripts were observed across both lymphoid and myeloid compartments. In addition, within the non-immune cell populations, *IDH3A* expression was detected in endothelial cells, epithelial cells, fibroblasts, and perivascular-like stromal cells without a single cell type showing uniquely dominant expression. To determine which of these *IDH3A*-expressing cell lineages contributes to the suppression of tumor growth, we examined the effect of *Idh3a* deletion in bone marrow and its derived cell populations. However, no significant change in tumor growth was observed under this condition. In contrast, *in vitro* co-culture experiments using MC38 cells and *Idh3a*-proficient or -deficient murine fibroblasts demonstrated that IDH3α in fibroblasts suppresses cancer cell proliferation. Furthermore, immunofluorescence analyses of MC38 transplanted tumors revealed no marked differences in intratumoral microvessel density or in the number of αSMA-positive fibroblasts infiltrating the tumor tissue, regardless of the *Idh3a* status of fibroblasts. Taken together, these findings suggest that IDH3α in fibroblasts may function in a tumor-suppressive manner. Nevertheless, given that single-cell RNA sequencing analysis revealed the presence of multiple non-malignant cell populations expressing IDH3A within tumor tissues, it cannot be concluded that IDH3α in fibroblasts alone is responsible for the observed tumor-suppressive effects. To directly address this question, it will be necessary to examine whether tumor growth is accelerated when *Idh3a* is specifically deleted in fibroblasts using mice expressing CreER^T2^ under the control of a fibroblast-specific promoter, such as *Col1a1, Col1a2*, and *Fsp1* (*S100a4*) [[26], [27], [28]].

In *in vitro* co-culture experiments using MC38 cancer cells and fibroblasts with different *Idh3a* statuses, we found that fibroblast IDH3α exerted a suppressive effect on cancer cell proliferation under high-density culture conditions that allow direct cell-cell contact. In contrast, when cancer cells were cultured in conditioned media derived from fibroblasts, the *Idh3a* status of fibroblasts did not affect cancer cell proliferation. Previous studies have established that metabolic reprogramming of CAFs toward aerobic glycolysis can promote tumor growth through the production and transfer of energy-rich metabolites, including lactate and pyruvate, which can subsequently be utilized by neighboring cancer cells [21]. Downregulation of IDH3α has been proposed to represent an important metabolic switch underlying this CAF phenotype [21]. Thus, our *in vivo* observation that stromal *Idh3a* deficiency promotes MC38 tumor growth is broadly consistent with the concept that loss of IDH3α can promote tumor-supportive functions of fibroblasts. However, our conditioned-medium experiments indicate that, under the experimental conditions used here, freely diffusible metabolites or other soluble factors alone are insufficient to reproduce the growth-promoting effect associated with *Idh3a*-deficient fibroblasts. Instead, the requirement for direct co-culture suggests that close-range metabolic coupling or other contact-dependent processes may be involved. One possible contact-dependent mechanism is the transfer of mitochondria with distinct functional properties from fibroblasts to cancer cells. Such mitochondrial transfer, which has recently attracted increasing attention [[29], [30], [31]], may arise from differences in *Idh3a* status and influence cancer cell proliferation. Further investigation of this possibility may provide mechanistic insight into the phenomena reported in this study.

## CRediT authorship contribution statement

**Lina Rochelle Koseki:** Writing – review & editing, Writing – original draft, Visualization, Validation, Methodology, Investigation, Funding acquisition, Formal analysis, Data curation. **Minoru Kobayashi:** Writing – review & editing, Methodology, Investigation, Funding acquisition. **Mai Ito:** Writing – review & editing, Resources, Investigation, Funding acquisition. **Yoko Nishiga:** Writing – review & editing, Resources. **Tamaki Saito:** Writing – review & editing, Investigation. **Shunji Haruna:** Writing – review & editing, Investigation. **Atsushi Shibata:** Writing – review & editing, Investigation. **Hiroshi Harada:** Writing – review & editing, Writing – original draft, Visualization, Validation, Supervision, Resources, Project administration, Methodology, Investigation, Funding acquisition, Formal analysis, Data curation, Conceptualization.

## Declaration of competing interest

These authors declare no competing interests.

## Appendix Supplementary materials

Image, application 1
