# Supplementary material for: IDH3-dependent mitochondrial function in stromal fibroblasts suppresses malignant tumor growth
Source: Neoplasia. 2026 Sep 4;81:101361. doi: 10.1016/j.neo.2026.101361 (PMC13572075; doi:10.1016/j.neo.2026.101361)
Supplement: Supplementary file 1 [file mmc1.pdf]

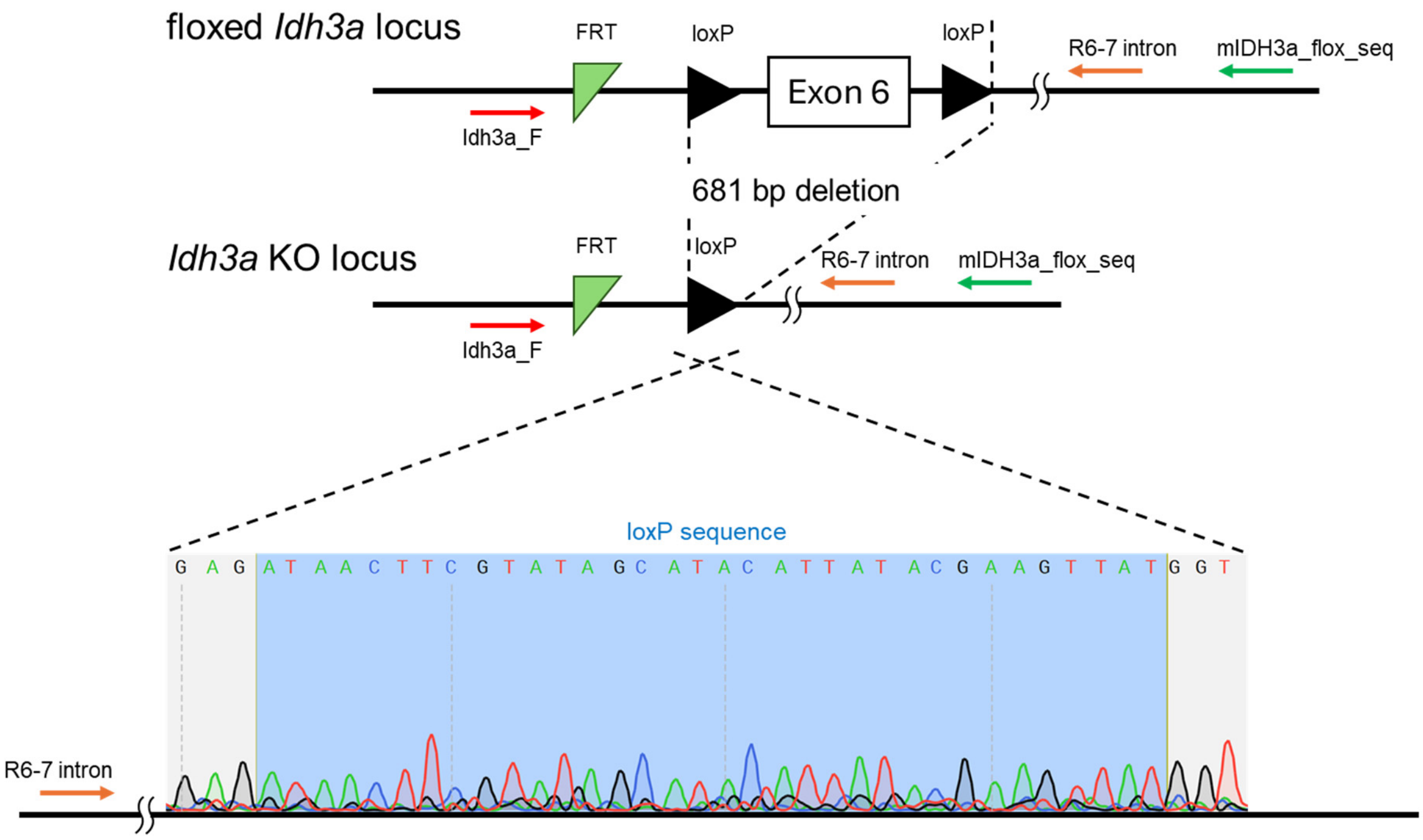

Supplementary Figure 1

Sanger sequencing analysis confirming Cre-mediated deletion of exon 6 at the *Idh3a* locus in *Idh3a* knockout (*Idh3a*$^{KO/KO}$; CreER$^{T2+/+}$) tail-tip fibroblast clones generated in this study. Sequencing data demonstrate precise recombination between loxP sites flanking exon 6, resulting in homozygous exon 6 deletion.

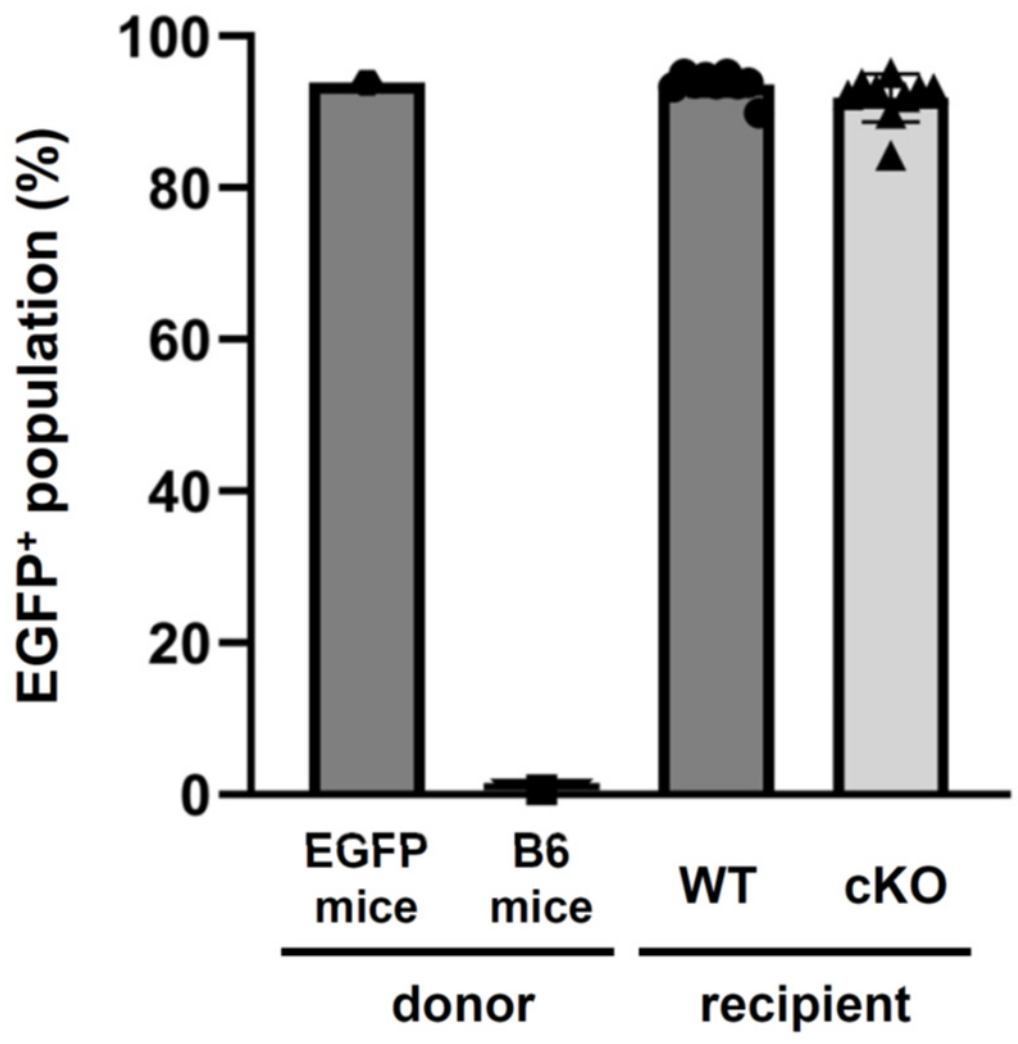

Supplementary Figure 2

FACS-based quantification of GFP-positive populations in bone marrow harvested from donor mice, C57BL/6-Tg(CAG-EGFP) mice (EGFP mice) or C57BL/6 mice (B6 mice) (donor: left two bars), and in peripheral blood harvested from recipient C57BL/6 mice 1 month after transplantation of bone marrow derived from EGFP-expressing C57BL/6-Tg(CAG-EGFP) mice (WT) or *Idh3a*$^{fl/fl}$; CreER$^{T2+/+}$; C57BL/6-Tg(CAG-EGFP) mice (cKO) (recipient: right two bars).

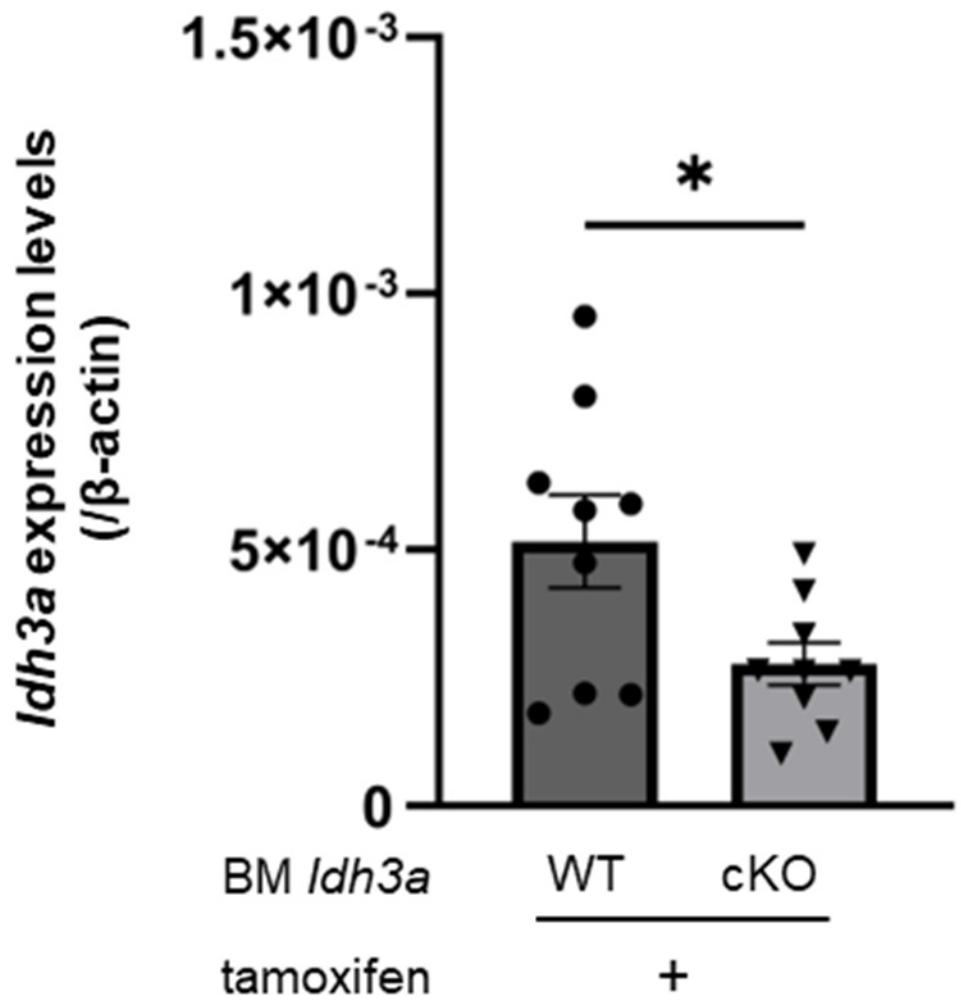

Supplementary Figure 3

qRT–PCR analysis of *Idh3a* mRNA expression using the same primer set as Figure 2 in peripheral blood of irradiated C57BL/6 recipient mice reconstituted with *Idh3a*$^{fl/fl}$; CreER$^{T2+/+}$; Tg(CAG-EGFP) (cKO) or C57BL/6-Tg(CAG-EGFP) (WT) bone marrow cells. Tamoxifen treatment (100 mg/kg/day for 5 consecutive days) was initiated 30 days after bone marrow transplantation (tamoxifen+), and peripheral blood samples were harvested 35 days after the initiation of tamoxifen administration. *Idh3a* mRNA levels were normalized to β-actin. Data represent mean ± SEM (n = 9 mice per group).
